# FROM POLICY TO PRACTICE: EVALUATING THE EFFECTIVENESS AND EXECUTION GAPS OF TLTCP 2.0 IN TAIWAN

**DOI:** 10.1093/geroni/igae098.3845

**Published:** 2024-12-31

**Authors:** Yu-Ting Chou, Kaichen Yao, Hsiu-Jung Wang, Ya-Mei Chen

**Affiliations:** National Taiwan University, Taipei City, Taipei, Taiwan (Republic of China); National Taiwan University, Taipei City, Taipei, Taiwan (Republic of China); Ministry of Health and Welfare Shuang-Ho Hospital, NEW TAIPEI CITY, Yunlin, Taiwan (Republic of China); National Taiwan University, Taipei City, Taipei, Taiwan (Republic of China)

## Abstract

Taiwan has implemented The Ten-Year Long-term Care Plan version 2.0 (TLTCP 2.0) for 7 years, focusing on integrated long-term care (LTC) services. Case managers at Community Integrated Service Center is the key and responsible for proposing managing service plan. However, gaps persist between policy and execution. To understand these gaps, related challenges, and outcomes, we conducted mixed-method study, including qualitative interviews with care managers and analysis of case managements outcomes using data from one a major center in northern Taiwan. Paired T-tests assessed differences between the Case Management Level (CML) scores at discharge and the first re-evaluation. Interviews revealed that the biggest gap and challenge is to become “one-stop shop.” TLTCP 2.0 users still face confusion due to multiple contacts during their service period. Case managers coordinated at least 140 cases monthly, yet the governments’ Care Services Management Information Platform is poorly designed to facilitate this coordination. Additionally, policy design flaws have led to service misuse, such as unclear requirement for range of motion services. Quantitative findings showed that while case mangers handle a higher caseload than in advanced countries, users demonstrate significant improvements in activities of daily living, primary caregivers’ burden, and CML scores after receiving integrated services. However, instrumental activities of daily living scores declined, likely due to reliance on service like house chores, leading to disuse these skills. Overall, the TLTCP 2.0 seems to be effective, but addressing policies and platform design and the issue of skill disuse is crucial for future improvement.

